# Naturalistic fMRI Language Mapping With Rotation Forest

**DOI:** 10.1002/hbm.70638

**Published:** 2026-09-11

**Authors:** Elaine Kuan, Viktor Vegh, John Phamnguyen, Kieran O′Brien, Amanda Hammond, David Reutens

**Affiliations:** ^1^ Centre for Advanced Imaging The University of Queensland Brisbane Australia; ^2^ Australian Institute for Bioengineering and Nanotechnology The University of Queensland Brisbane Australia; ^3^ ARC Training Centre for Innovation in Biomedical Imaging Technology The University of Queensland Brisbane Australia; ^4^ School of Human Movement and Nutritional Sciences The University of Queensland Brisbane Australia; ^5^ Siemens Healthcare Pty Ltd Melbourne Australia; ^6^ Royal Brisbane and Women's Hospital Brisbane Australia

**Keywords:** language, movie watching, naturalistic fMRI, naturalistic stimulus

## Abstract

Naturalistic fMRI offers a patient‐friendly alternative to task‐based language mapping but presents analytical challenges due to complex brain responses. This study presents an automated framework for language mapping using naturalistic fMRI analysed with a Rotation Forest (RotF) classifier. Participants were presented a televised quiz show during fMRI acquisition and completed seven task‐based language paradigms, which were used as reference maps. RotF models were trained on voxel‐wise time series labelled using task‐based activation maps and evaluated on independent participants. Performance was assessed using the structural similarity index measure (SSIM) and the language lateralisation indices (LI). RotF generated activation maps demonstrated strong spatial and lateralisation agreement with task‐based activation, capturing both receptive and expressive language regions. Data quality, which was assessed through head motion and post‐scan questionnaire score, influenced model performance and provided a practical quality control measure. Feature selection identified a short, informative stimulus segment, enabling reliable language mapping with a scan duration of approximately 5 min. These findings demonstrate the feasibility of accurate, efficient and patient‐friendly language mapping using naturalistic fMRI combined with machine learning.

## Graphical abstract

This study presents an automated framework for language mapping using naturalistic fMRI analysed with a Rotation Forest (RotF) classifier. The approach produces activation and lateralisation patterns comparable to task‐based fMRI and remains effective with a shortened 5‐min stimulus, supporting efficient presurgical language assessment.

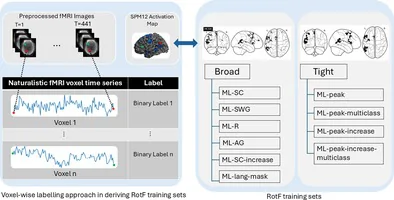

## 1 Introduction

Language mapping is a procedure undertaken by clinicians or researchers to identify regions of the brain involved in language processing. Language lateralisation and localisation vary in normal subjects and can also be re‐organised in neurological disease (Seghier et al. 2004; Knecht et al. 2003; Berl et al. 2014; Powell et al. 2007). As a result, accurate localisation of language function in the cerebral cortex is clinically important in patients undergoing neurosurgical procedures that could threaten the eloquent cortex. It impacts decisions about the feasibility of surgery, surgical planning and intra‐operative surgical guidance. Accurate lateralisation and localisation of key areas for language processing are also important to prevent post‐operative deficits (Benjamin 2020).

Functional Magnetic Resonance Imaging (fMRI) is a non‐invasive and safe neuroimaging tool that is commonly used to map human brain activation by cognitive processes such as language (Benjamin 2020; Bookheimer 2007). Language mapping using fMRI is conventionally performed with task‐based fMRI, involving specially designed stimuli to activate specific brain responses. In contrast, naturalistic fMRI, an emerging approach for functional language mapping in patients, utilises dynamic video stimuli encountered in daily life such as movies (Sonkusare et al. 2019; Spiers and Maguire 2007; Jääskeläinen et al. 2021). Naturalistic fMRI offers potential advantages because patients are not required to understand and perform unfamiliar in‐scanner tasks and movie watching may be more engaging for patients (Vanderwal et al. 2019), bringing the positive benefit of enhanced fMRI time series signal properties leading to temporal signal‐to‐noise ratio (tSNR) improvements (Aliko et al. 2020; Eickhoff et al. 2020; Hasson et al. 2010; Finn and Bandettini 2021). In a clinical setting, naturalistic fMRI may be less burdensome to implement than task‐fMRI because it does not require technical understanding of fMRI presentation software to deliver time‐stamped tasks, and patients do not require fMRI task training before entering the scanner.

Despite growing interest in streamlining clinical workflows, there are limited studies on the use of naturalistic fMRI in mapping language regions of individual subjects. One of the major challenges associated with naturalistic fMRI is the complexity of analysing such data. Meaningful regressors may be difficult to define as naturalistic stimuli may induce complex brain responses. Commonly used approaches to this problem include Independent Component Analysis (ICA) and Inter‐subject or Intra‐subject correlation (ISC), which aim to identify similarities in brain responses between participants in a population rather than analysing activation in individuals (Hasson et al. 2010; Bartels and Zeki 2004; Lahnakoski et al. 2012; Kauppi et al. 2017; Simony and Chang 2020). Multi‐voxel Pattern Analysis (MVPA) and machine learning methods have been used to decode stimuli from brain responses (Norman et al. 2006; Vodrahalli et al. 2018; Khosla 2020; Oota 2019), but their use has primarily focused on brain‐computer interface‐like applications. Application of these methods to mapping of brain activation is scarce.

The research herein aims to describe the potential of the Rotation Forest (RotF) algorithm to identify language activation and develop an automated approach for language mapping based on naturalistic fMRI data. RotF is chosen as it has been shown to produce language activation maps from task‐based fMRI data similar to those generated using the conventional methods of fMRI time series analyses (Kuan et al. 2024). Based on this foundation, we hypothesised that the method, when applied to naturalistic fMRI data, produces language activation maps alike those obtained using task‐based fMRI data and conventional time series pattern analysis.

## 2 Materials and Methods

A tailored televised quiz show was presented to recruited participants to acquire naturalistic fMRI data. In addition, participants completed a battery of language task fMRI scans in the same MRI scan session. With the acquired naturalistic and task‐based fMRI data, a number of training sets were constructed, and RotF models were trained and tested. Information regarding participant recruitment, naturalistic and task stimulus design, RotF training and test sets and performance evaluation measures are outlined below.

As the characteristics of an effective naturalistic stimulus for reliable language activation maps are unknown, the Sequential Forward Feature Elimination (SFFE) feature selection technique was applied to identify quiz show segments most relevant in language activation within individuals. With improved clinical workflows in mind, a shortened segment of the naturalistic fMRI sequence was identified and tested to understand plausible naturalistic fMRI scan times.

### 2.1 Participants

Multiple related studies were approved by the Royal Brisbane and Women's Hospital Human Research Ethics Committee (RBWH Human Research Ethics Committee ethics approval number: HREC/2021/QRBW/74461). A total of 20 healthy participants without a history of brain disorders or injury (mean age 39 years, range 21–71 years, 10 females; recruited via targeting advertising) and 30 individuals with epilepsy (mean age 39, range 19–67 years, 15 females; recruited through the Neurology Department of the Royal Brisbane and Women's Hospital) participated in the study. Study participants were required to have English as their primary language, and handedness was assessed using the Edinburgh Handiness Inventory (EHI) Questionnaire. All participants provided written informed consent. While there is no specific clinical decision making reason for the inclusion of patients, we opted to append our existing cohort simply to understand patient compliance and to increase the heterogeneity and size of the dataset necessary for robust training and testing of RotF.

### 2.2 Reference Language Activation Maps

Participants completed seven block‐designed task‐based language fMRI studies with paradigms assaying Sentence Completion (SC), Silent Word Generation (SWG), Rhyming (R), Object Naming (ON), Antonym Generation (AG), Passive Story Listening (PSL), and Sentence Completion Listening (SCL). The task stimuli were presented with E‐prime 3.0 software (Psychology Software Tools Inc., version 3.0).

### 2.3 fMRI Data Acquisition

Data were collected using a Siemens Magnetom 3 T Prisma scanner (Siemens Healthcare, Erlangen, Germany) installed at the Herston Imaging Research Facility, Brisbane, Australia. Blood Oxygen Level Dependent (BOLD) task activated functional MRI scans were acquired using an Echo Plannar Imaging (EPI) sequence and a standard 64 channel head coil (TR = 2000 ms, TE = 23 ms, flip angle = 90°, in‐plane matrix size = 70 × 70, 42 axial slices, voxel size = 3 × 3 × 3 mm^3^). Whole brain T1 MPRAGE structural images were also acquired (TR = 1900 ms, TE = 256 ms, flip angle = 9°, in‐plane matrix size = 256 × 256, 192 axial slices, voxel size = 1 × 1 × 1 mm^3^).

### 2.4 Pre‐Processing of fMRI Data

All functional data were pre‐processed using Statistical Parametric Mapping SPM12 software (https://www.fil.ion.ucl.ac.uk/spm/software/spm12/). The pre‐processing steps included slice timing correction, realignment (to mean image functional image across all tasks for a single participant), co‐registration to structural T1 image, spatial normalisation to Montreal Neurological Institute (MNI) space (re‐sampled to 3 × 3 × 3 mm^3^) and smoothing using a Gaussian kernel of 6 mm full‐width half maximum (FWHM). Voxels activated by the task were considered at the p<0.001 level.

### 2.5 Naturalistic fMRI Experiment

#### 2.5.1 Naturalistic Stimulus Content

The naturalistic stimulus is derived from an episode of Millionaire Hot Seat (2017 Episode 55, Australian version), a popular late afternoon quiz show. This naturalistic stimulus was chosen as it is rich in spoken and visual language components, including questions and answers, which elicit language activation. Quiz shows are also engaging, entertaining, and family‐friendly, together making such content an appealing choice for participants and patients.

The quiz show was edited to include advertisement segments, replicating an actual TV show. It was also broken into shorter segments of 1–5 min in duration with 1 min advertisement segments in between quiz questions. A total of four quiz questions were interleaved with three advertisement segments. The advertisement segments consisted of tourism or promotional videos taken from YouTube, and did not contain spoken language. Only occasional written words were present in the advertisement segments. The total duration of the video clip was 14 min and 52 s. Participants were given MRI‐safe active noise‐cancelling headphones, and those who required vision correction were encouraged to wear contact lenses or provided with MRI‐safe vision correction lenses. In addition, participants were instructed to stay still and pay attention to the quiz show. No additional instructions were provided to ensure that the experience remained as natural as possible.

#### 2.5.2 Naturalistic fMRI Time Series Data Collection

Data for the naturalistic fMRI experiment were obtained according to the task fMRI protocol without triggering based on the stimuli. This led to time series data collected with a 2 s interval between image volumes. The naturalistic stimulus was started at the start of the quiz show, resulting in 446 time series data points. The time series were labelled manually for segments corresponding to advertisement and segments with quiz questions.

#### 2.5.3 Measure of Participant Attention and Head Motion

Each participant completed a post‐scan test containing questions relating to the quiz show questions, which served as an indirect measure of attention. Participants were awarded 1 point for each question they answered correctly. For questions containing two sections, participants were awarded a score of 0.5 for each part of the question answered correctly. The highest score achievable on the test was 6 points. Head motion for each individual in each language paradigm was accessed using Framewise Displacement (FD) (Power et al. 2012). Using questionnaire scores and head motion estimates, the impact of these factors on naturalistic fMRI activation maps was considered.

### 2.6 RotF Training and Test Pipeline

Based on previous work, RotF was established as the best performing method in the context of voxel‐wise language task‐based fMRI analysis and produced robust language activation maps when compared to other categories of time series classification methods including deep learning methods (Kuan et al. 2024). Therefore, distinct RotF models were trained using the various training data to perform the task of classification on the extracted 1D naturalistic fMRI time series using the Python implementation from SKTIME (version 13.4) (Löning 2019; SKTIME 2023). In view of the training sets used, and their particular differences, optimisation of hyperparameters would confound the understanding of how the training set should be constructed. For this reason, hyperparameters were left as the default values. To understand how different voxel labelling strategies for the naturalistic fMRI time series dataset affect RotF training, a series of training sets were constructed and evaluated using independent test sets.

#### 2.6.1 Training

Training sets were constructed using the pre‐processed naturalistic fMRI time series data of 14 healthy participants, consistent with methodology in (Kuan et al. 2024). Data from healthy participants were selected to construct the training sets due to their consistency and well‐characterised brain activation patterns, which made them a reliable reference for model development and evaluation.

Each participant's naturalistic fMRI data were labelled with voxel labels from their task activation maps. Binarised task‐based activation maps for each individual were derived using the Statistical Parametric Mapping (SPM12) software, following the steps described in (Kuan et al. 2024). As the individual task or naturalistic fMRI time series dataset of each participant was co‐registered to their structural T1, the number of voxels in each activation map directly corresponded to the number of voxel‐time series in any of that participant's datasets, including the naturalistic paradigm dataset. This enabled the labelling of participant specific naturalistic fMRI time series with their corresponding task activation maps.

Unless otherwise specified, a random sampling procedure was used to construct the training sets. In this procedure, a subset of the naturalistic fMRI voxel time series dataset was selected randomly to create the final training sets. The number of naturalistic fMRI voxel time series sampled from each participant's data and the number of voxels labelled as 0 or 1 was kept the same in each training set. For example, if a total of 4872 spatial locations from which times series samples were taken for the training set, a random sample of 174 naturalistic fMRI time series from voxels labelled with 1 (‘activated’) and 174 time series from voxels labelled with 0 (‘non‐activated’) was used from each of the 14 participants' naturalistic fMRI scans with locations chosen according to the their individual task‐based fMRI activation maps.

**FIGURE 1:**
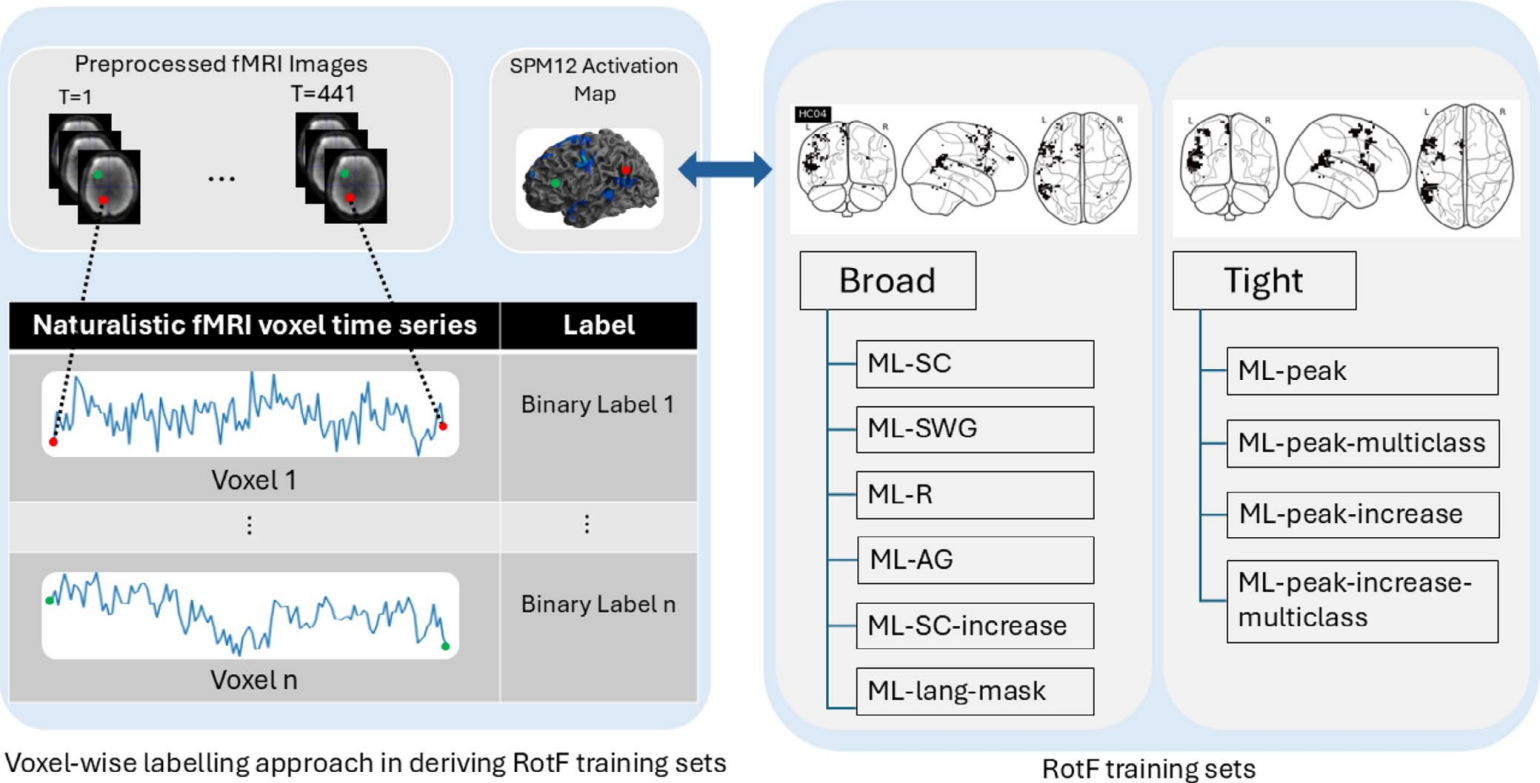
Illustrated are the steps in creating voxel‐wise naturalistic fMRI time series training sets. The nomenclature covers the cases of Broad and Tight sampling schemes for RotF training, respectively capturing random sampling within regions of language activation (i.e., SC, SWG, R, AG) versus tight sampling around the ‘peak’ of activation. Table 1 provides the details of the various tests.

There were two categories of choosing activated voxels for the training sets: 1. Broad—random samples were acquired from the whole activated region in the task‐based fMRI activation maps, and 2. Tight—random samples were drawn from voxels close to activation peaks. In either case, time series data corresponding with no activation were sampled randomly from the set of non‐activated voxels irrespective of distance to any activation peak. The process of creating training sets and the different training sets are illustrated in Figure 1.

Table 1 summarises each of the task activation maps used for labelling the naturalistic fMRI time series to create training datasets. In addition to individual task activation maps, a combined task activation map for each participant was also derived by adding binarised task activation maps from the seven language tasks. The combined task activation map was included to increase the strength of activation peaks and used to guide the construction of training sets in the ‘Tight’ category.

**TABLE 1:** Summary of task activation maps used to construct RotF training sets.

Name	Description	Samples
ML‐SC	SC activation maps were used.	4872
ML‐SWG	SWG activation maps were used.	4872
ML‐R	R activation maps were used.	4872
ML‐AG	AG activation maps were used.	4872
ML‐SC‐increase	SC activation maps were used with quadruple number of training samples from each participant.	19,488
ML‐lang‐mask	Training using functional language regions of interests idenitified by Fedorenko et al. and SC activation maps (Fedorenko et al. 2010).	4872
ML‐peak	Training using combined task activation map but sampling close to the peak. All seven task activation maps of each participant were binarised and fused to form a combined task activation map.	4872
ML‐peak‐multiclass	Training using combined task activation map thresholded according to the procedure described in ML‐peak with occipital activation and language activation separated into their own classes.	7308
ML‐peak‐increase	Training using combined task activation map described ML‐peak with a less stringent task activation threshold (the top 70% values were considered activated voxels) to increase number of activated samples.	8962
ML‐peak‐increase‐multiclass	Training using combined task activation map but with occipital activation and language activation as separate classes.	17,856

#### 2.6.2 Testing

The test involved predicting whether each time series sequence corresponding to a specific spatial brain location was activated in the 36 participants not used for training the RotF model. On average, participants had 63,234 time series samples across their brains. When extracting the time series data, the location was saved as well before feeding each 1D time series into the trained RotF. Using the spatial coordinate, the RotF prediction was assigned to a location in the participant's brain. When repeated for all voxels in the brain, a spatially resolved map of RotF predicted activation was produced for each test case individual.

### 2.7 Evaluation of RotF Predicted Language Activation

The Structural Similarity Index Measure (SSIM), Dice Coefficient (DICE) and Lateralisation Indices (LI) were used to assess the performance of RotF trained on the different training sets listed in Table 1. SSIM and DICE assess how well RotF reconstructs the spatial structure of activation maps, capturing language cluster shape, contrast and overall similarity between activation maps. In contrast, LI assesses hemispheric dominance, a key clinical feature of language fMRI. Together, these two measures provide insights on the overall spatial patterns and accuracy of language lateralisation patterns of the RotF activation maps, which are crucial in language mapping.

#### 2.7.1 Correspondence Between RotF and Task fMRI Activation Maps

SSIM and DICE coefficients between each test participant's RotF generated activation map and their corresponding seven language task activation maps were assessed. These comparisons were performed within subjects and within brain regions for each RotF generated activation map. For each comparison (e.g., ML‐SC vs. SC), the SSIM or DICE values were averaged across test participants to produce group level data. The results were visualised using box plots, showing the distribution of averaged SSIM and DICE coefficient values between a RotF generated activation map and the seven language task activation maps.

The Appendix provides the steps for post‐processing of activation maps and SSIM calculation. Prior to SSIM calculation, both language task and RotF generated activation maps were normalised to 1. SSIM values can take values between 0 and 1, with 1 indicating a perfect correspondence between activation maps.

A Friedman test with pairwise Wilcoxon signed‐rank test (*p*‐value corrected for multiple comparisons using the Benjamini‐Hochberg method) was performed to identify statistically significant differences between the participant‐level SSIM values. Statistical analyses were conducted on raw participant‐level SSIM values rather than aggregate means to preserve within‐subject variability and ensure appropriate statistical inference. Statistical significance was assumed at *p* < 0.05.

#### 2.7.2 Language Activation Lateralisation

The Laterality Index (LI) was calculated for the frontal and temporal regions as a measure of language lateralisation. Masks for the frontal and temporal regions were derived from the WFU PickAtlas toolbox, an extension of the Statistical Parametric Mapping software. Prior to LI analysis, auditory activation were excluded by masking out the auditory cortices using the Neurosynth auditory mask obtained from Neurovault (Gorgolewski et al. 2015). LI analysis were performed at different activation thresholds (0.1, 0.15, 0.2, 0.25, 0.3). Laterality was calculated using the formula LI $=\frac{Q_{L}-Q_{R}}{Q_{L}+Q_{R}}$, where $Q_{L}$ denotes the number of voxels with a value above the specified threshold in the left‐sided region of interest and $Q_{R}$ the number of voxels with a value above a specified threshold in the homologous right‐sided region of interest (Hinke et al. 1993; Desmond et al. 1995; Binder et al. 1996). LI values range from −1 to 1, and LI values exceeding 0.2 were taken to indicate left hemisphere dominance, values less than −0.2 to indicate right hemisphere dominance and a values between −0.2 and 0.2 indicate bilaterality, in keeping with the LI thresholds defined by (Tie et al. 2015; Yao et al. 2022). Perfect concordance of lateralisation would indicate similar lateralisation between the RotF activation maps and task activation maps.

### 2.8 Naturalistic fMRI Time Series Feature Importance

Sequential Forward Feature Elimination (SFFE) (Ferri 1994) was used to identify the most informative features within a dataset by iteratively evaluating the impact of an increasing number of features on the cross‐validation performance of a machine learning model. The algorithm begins with an empty feature set and iteratively adds features until no additional features provide a meaningful performance gain or a predefined stopping criterion is reached.

Here, SFFE was applied to identify the features (time frames) contributing most to the prediction by RotF trained using the ML‐SC training set. The ML‐SC training set was chosen because the sentence completion task is known to elicit robust language activation and is recommended by the American Society of Functional Neuroradiology as the first choice among tasks used for presurgical language mapping (Black et al. 2017). As the ML‐SC training set is composed of a set of 1D fMRI time series with time adjacency dependence (note, BOLD contrast is on the order of 5 s, whereas time points are separated by 2 s), the SFFE method identifies the collective contribution of the set of fMRI time points to the RotF performance.

SFFE returns a binary 1D vector denoting important time frames as 1. The stopping criterion for this analysis was 10 features, as SFFE is computationally expensive. To overcome limitations of this stopping criterion and to improve robustness, SFFE is repeated 5 times on the same training set and model, resulting in 5 binary vectors. The 5 binary vectors were added to produce a feature importance waveform. Higher spikes highlight a higher contribution from the time segment. This analysis was the basis for defining a shortened version of the naturalistic fMRI stimulus.

## 3 Results

### 3.1 RotF With All 1D Naturalistic fMRI Time Points

#### 3.1.1 Correspondence Between RotF and Task fMRI Activation Maps

**FIGURE 2:**
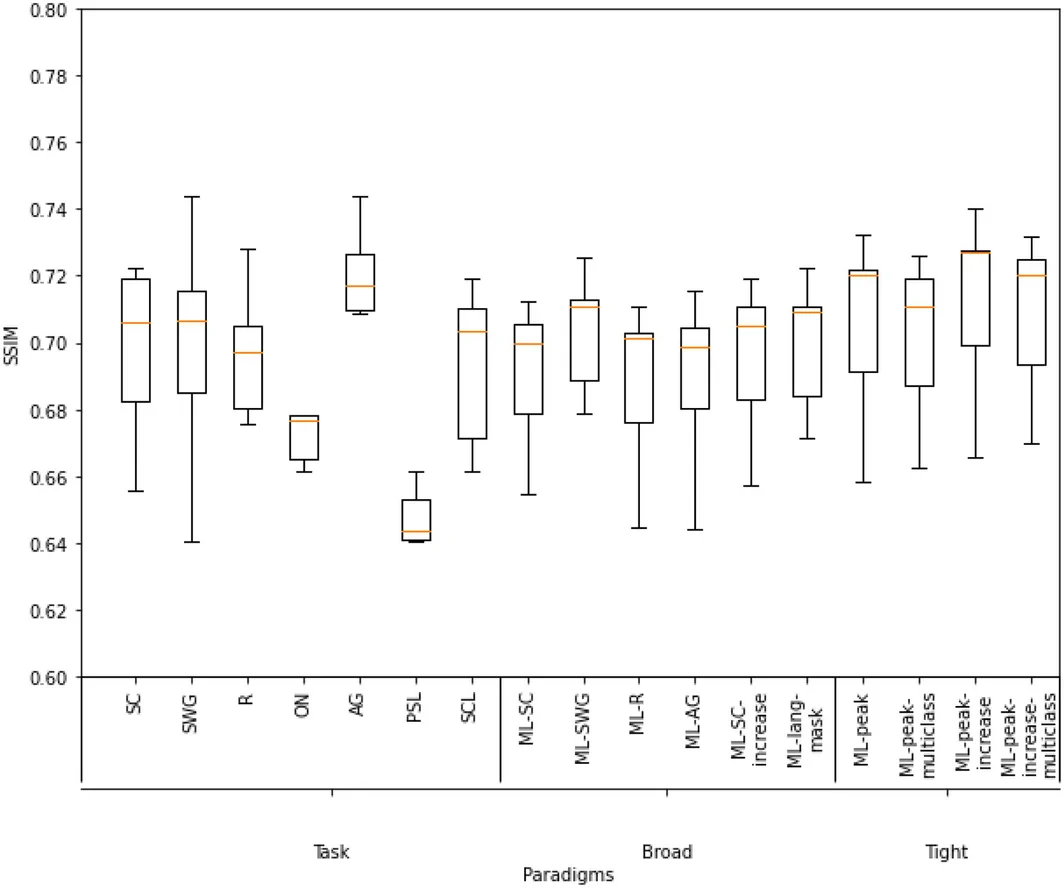
Box plot of mean SSIM of each paradigm organised as task and RotF training scenarios using broad and tight sampling versus seven task paradigms across all test participants. Participant‐level SSIM differed across paradigms, with the R, ON and PSL tasks significantly lower than the SC, SWG, AG and SCL tasks. The AG task had significantly larger SSIM than the other tasks. The ON and PSL tasks had significantly smaller SSIM than all RotF tested cases; other differences were not identified.

Figure 2 shows the box plots which represent the distribution of mean SSIM values when the activation maps of paradigms described in the x‐axis were compared to the different language task activation maps. The distribution of mean SSIM values ranged between 0.63 and 0.74 across the different paradigms. PSL had the lowest overall median, suggesting a low correspondence between the PSL and remaining tasks. The distribution of mean SSIM values exhibited limited variability across the different RotF training scenarios, as observed by the overall median (the orange line) in the box plot. The ML‐peak‐increase training scenario had the highest overall median SSIM across the different training scenarios.

The participant‐level SSIM differed significantly between the paradigms (Friedman Test, $H\left(16\right)=684,p=0$). The post hoc Wilcoxon signed‐rank tests revealed no significant differences between the participant‐level SSIM of the SC, SWG and SCL tasks. The participant‐level SSIM of the R, PSL and ON task was significantly smaller than of the four tasks (SC, SWG, AG and SCL) while the AG task was significantly larger than all the tasks. The ML‐peak‐increase and ML‐peak‐increase‐multiclass scenario showed significantly larger participant‐level SSIM compared to six tasks except for AG for which there was no statistical significance. The AG task showed significantly larger participant‐level SSIM when compared to the remaining ML training scenarios. The ON and PSL tasks had significantly smaller participant‐level SSIM than all RotF training scenarios. A significant difference in participant‐level SSIM was not found between the SWG, R, SCL tasks and the ML‐SC, ML‐SWG and ML‐increase training scenarios. Overall, the ML‐peak‐increase training scenario had the largest participant‐level mean SSIM and was investigated further.

**FIGURE 3:**
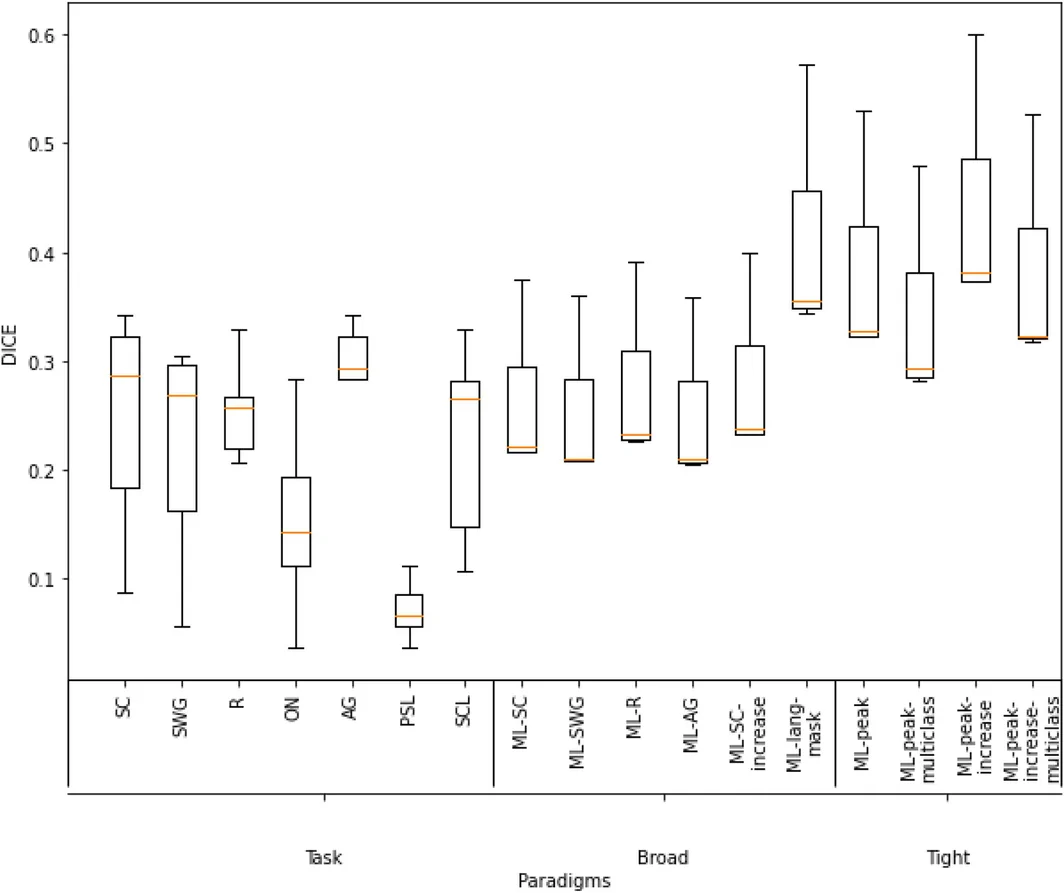
Box plot of mean DICE of each paradigm organised as task and RotF training scenarios using broad and tight sampling versus seven task paradigms across all test participants.

Figure 3 shows box plots which represent the distribution of mean DICE when the activation maps of paradigms described in the *x*‐axis were compared to the different language task activation maps. For the language tasks, the distribution of mean DICE follows the trends similar to the SSIM. However, more variation was present with the RotF training scenarios, with RotF training scenarios of the ‘Tight’ category outperforming the other RotF training scenarios and task paradigms. The ML‐peak‐increase training scenario also had the highest overall median DICE across the different training scenarios.

#### 3.1.2 Effect of Head Motion and Score on Post‐Naturalistic fMRI Questionnaire

**FIGURE 4:**
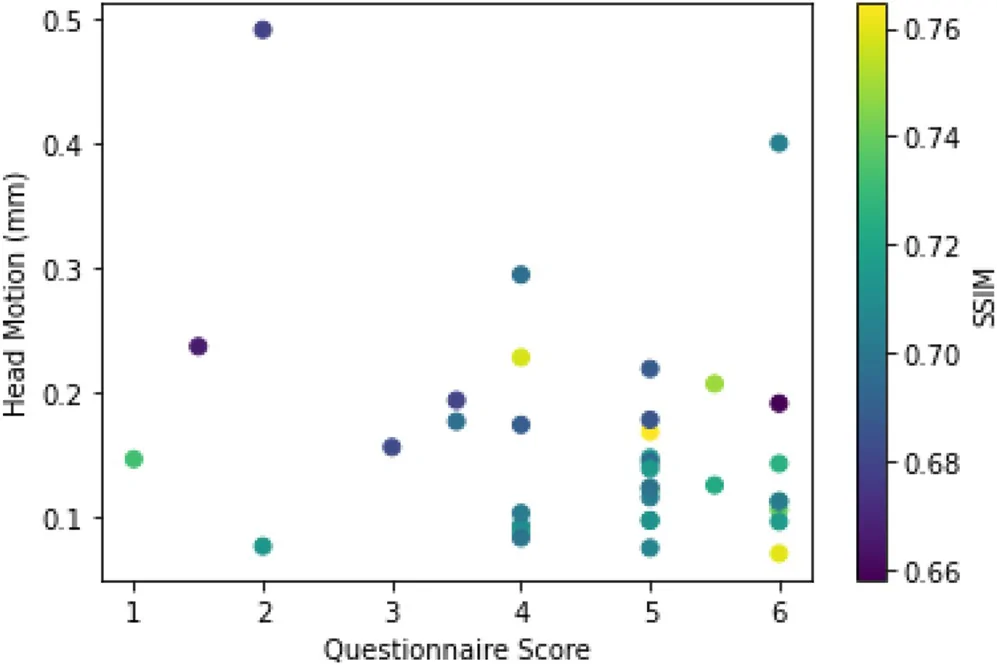
Scatter plot of head motion versus questionnaire score for all test participants, colour‐coded by mean SSIM of the ML‐peak‐increase training scenario.

Figure 4 plots the relationship between head motion and questionnaire scores for each participant. Each point is colour‐coded based on an average SSIM value, calculated by comparing activation maps from the ML‐peak‐increase training scenario with those from the seven task paradigms. From Figure 4, SSIM larger than 0.72 occurred when the questionnaire score was 4/6 or higher and when motion was less than 0.23 mm.

#### 3.1.3 Language Activation Lateralisation

LI analysis was performed on participants who achieved a questionnaire score ≥ 3 and head motion ≤ 0.2 mm (Cut‐off 1) and on participants who achieved a questionnaire score ≥ 4 and head motion ≤ 0.1 mm (Cut‐off 2). Opposite laterality between two activation maps was used to define dissimilarity. The activation threshold(s) which gave the lowest percentage of participants with dissimilarity were reported, which can be found in Table 2.

**TABLE 2:** This table shows the activation threshold(s) for which the lowest percentage of participants that present with laterality index dissimilarity between the activation maps produced by the ML‐peak‐increase training scenario and seven task activation maps.

% (Threshold)	Frontal, cut‐off 1	Frontal, cut‐off 2	Temporal, cut‐off 1	Temporal, cut‐off 2
SC	19 (0.25)	13 (0.25, 0.3)	33 (0.15, 0.2)	13 (0.15, 0.2, 0.25, 0.3)
SWG	30 (0.3)	38 (0.15, 0.2, 0.25)	33 (0.3)	25 (0.2, 0.25, 0.3)
R	33 (0.3)	25 (0.3)	30 (0.3)	13 (0.3)
ON	41 (0.1, 0.2, 0.25, 0.3)	25 (0.15, 0.2, 0.25, 0.3)	30 (0.3)	13 (0.3)
AG	30 (0.1)	13 (0.3)	41 (0.15, 0.2)	25 (0.2, 0.25, 0.3)
PSL	41 (0.3)	25 (0.3)	44 (0.1)	38 (0.15, 0.2, 0.25, 0.3)
SCL	33 (0.3)	25 (0.1,0.25,0.3)	41 (0.25, 0.3)	25 (0.15, 0.2, 0.25, 0.3)

*Note:* Laterality index dissimilarity was computed for the frontal and temporal ROIs and aggregated according to (i) Cut‐off 1: Head motion and questionnaire score cutoff of 0.2 mm and 3; and (ii) Cut‐off 2: Questionnaire score cut off of 0.1 and 4.

The identified thresholds varied substantially between tasks and ROIs, indicating that the optimal activation level for minimising LI dissimilarity is task and region‐dependent. In general, a lower dissimilarity percentage is observed in temporal ROIs at higher activation thresholds compared to frontal ROIs, especially in Cut‐off 1. In contrast, applying the stricter criteria of Cut‐off 2 resulted in higher variability, and when compared to several tasks the minimum dissimilarity percentages were observed to increase, suggesting reduced robustness under more stringent filtering conditions. The PSL, AG, and SCL tasks demonstrated relatively higher minimal dissimilarity percentages in temporal regions, whereas others (e.g., SC and SWG) showed comparatively lower values in frontal regions, highlighting the differences in task activations.

#### 3.1.4 Examples of RotF Predicted Activation Maps

**FIGURE 5:**
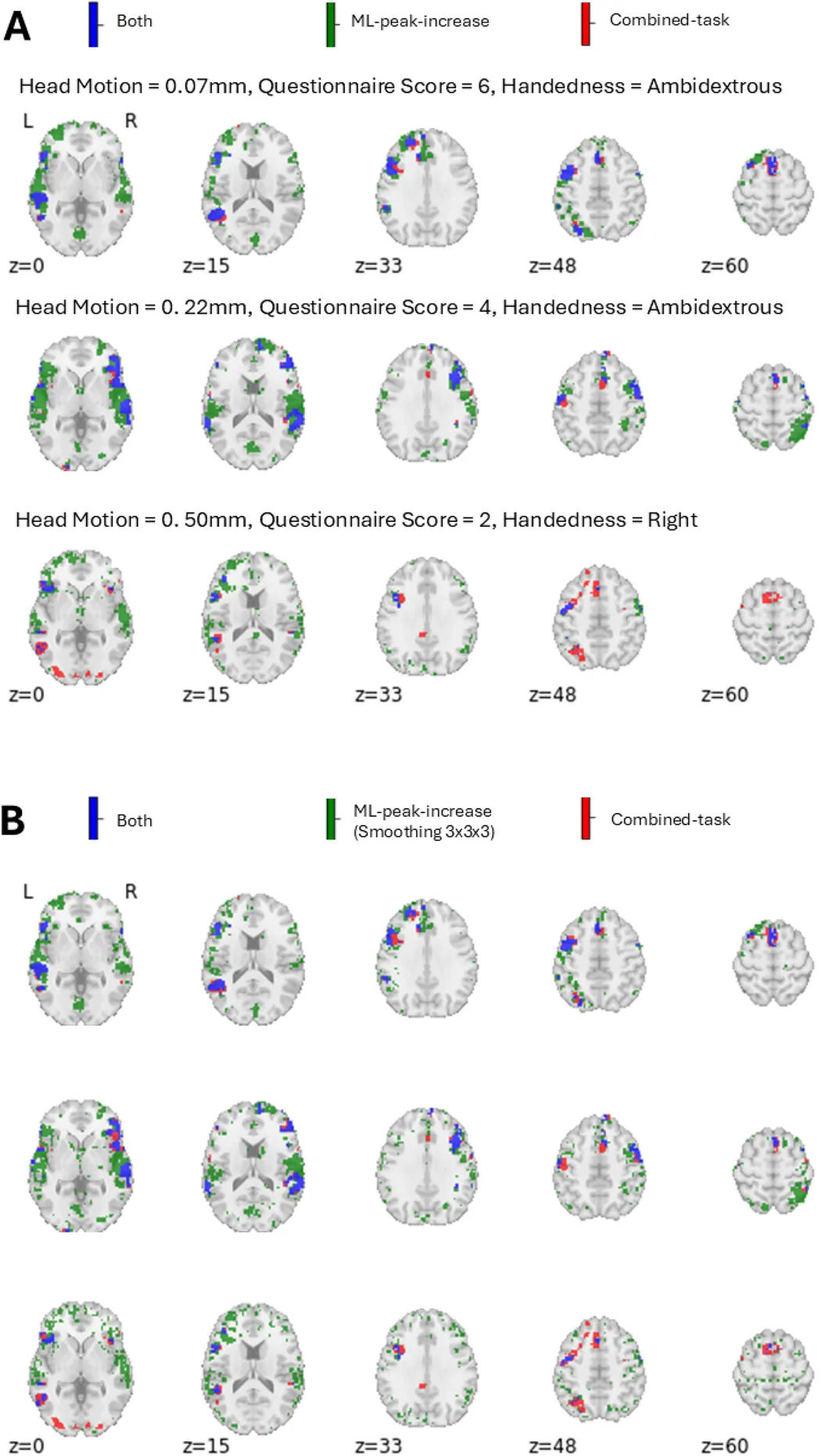
This figure shows the overlap between the combined task activation map versus the activation maps generated by the ML‐peak‐increase training scenario (A) and ML‐peak‐increase training scenario with reduced smoothing (B) of three participants (Blue—Overlap, Green—ML‐peak‐increase, Red—Combined‐task).

Figure 5 shows the activation maps produced by the ML‐peak‐increase training scenario and the ML‐peak‐increase training scenario with reduced spatial smoothing (3 × 3 × 3 kernel compared to the initial 6 × 6 × 6). The activation maps were superimposed on corresponding axial slices from the MNI brain template. A combined task activation map comprising the sum of all task activation maps has also been overlaid. A threshold of 0.2 was applied to both activation maps for illustrative purposes. The group of three rows are aggregated according to three test participants who performed best, moderately well and worst in terms of questionnaire score and motion (top, middle and bottom row).

Blue areas denote the overlap between the ML‐peak‐increase and the combined task activation maps, green areas denote areas activated only in the ML‐peak‐increase training scenario and red areas denote areas activated only in the combined task map. Language clusters, including those in the inferior frontal gyrus and posterior superior temporal gyrus, are captured by all three RotF activation maps but the degree of overlap varies between participants. Bilateral temporal activation is seen in the RotF activation map of all three participants, reflecting the auditory components in the naturalistic fMRI paradigm. Questionnaire score and head motion had an impact on the RotF activation maps. The participant with the highest questionnaire score and lowest head motion (top row of Figure 5) had well defined language activation clusters in both the frontal and temporal regions of the brain, which overlapped well with the task activation map. While activation was seen in the inferior frontal gyrus for the participant with low questionnaire score and high head motion, the activation cluster is less prominent. Clusters of activation in the posterior and superior frontal regions were not captured by RotF for this participant. Similar activation patterns were observed in the activation maps with reduced smoothing, suggesting that the RotF model is robust to reduced smoothing. While not shown here, ML‐peak‐increase activation maps with no smoothing also exhibit similar activation clusters; however, more activation specks are noticed.

### 3.2 Importance of Naturalistic fMRI Time Frames

**FIGURE 6:**
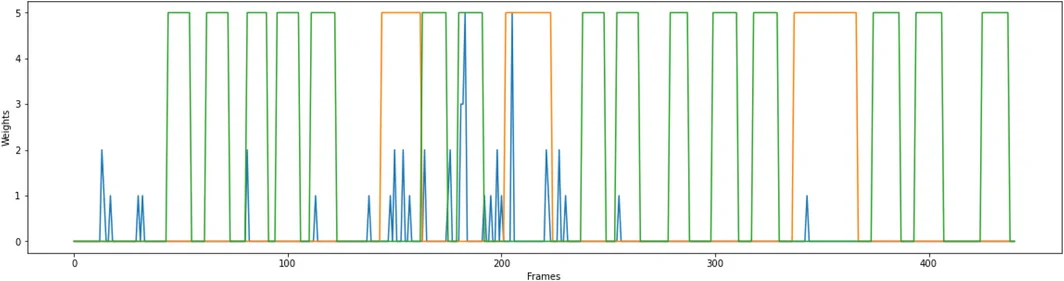
This figure shows the SFFE waveform averaged across the 5 SFFE runs. The blue plot shows the weighting for time frame importance. The orange shows the time frames corresponding to advertisement segments and the green blocks are the frames over which questions were shown during the quiz show (i.e., in the naturalistic fMRI stimulus).

We observe in Figure 6 that the SFFE identified spikes in the blue waveform condensed around time frames 130 to 260 (specifically 138–255, length of 117 frames), with largest spikes between 190 and 210. The concentration of spikes indicated the importance of distinct frames in predicting activation using the RotF model. The RotF model was then retrained and re‐evaluated with naturalistic fMRI time series segments of length 117 frames. Three different segments were investigated: time frames 0 to 117, 138 to 255 and 324 to 441.

### 3.3 RotF Applied to the Shortened Naturalistic fMRI Time Series

RotF models were trained and evaluated on three shortened naturalistic fMRI time series segments using the same voxel sampling procedure for the ML‐SC or ML‐peak‐increase. They are referred to as ML‐SC_0–117, ML‐SC_138–255, ML‐SC_324–441 and ML‐peak‐increase_0–117, ML‐peak‐increase_138–255 and ML‐peak‐increase_324–441. Whilst SFFE was performed only on the RotF model trained using the ML‐SC training scenario, the ML‐peak‐increase was also retrained and evaluated using the three segments as it performed the best among the different RotF training scenarios.

#### 3.3.1 Similarity Between Language Activation Maps

**FIGURE 7:**
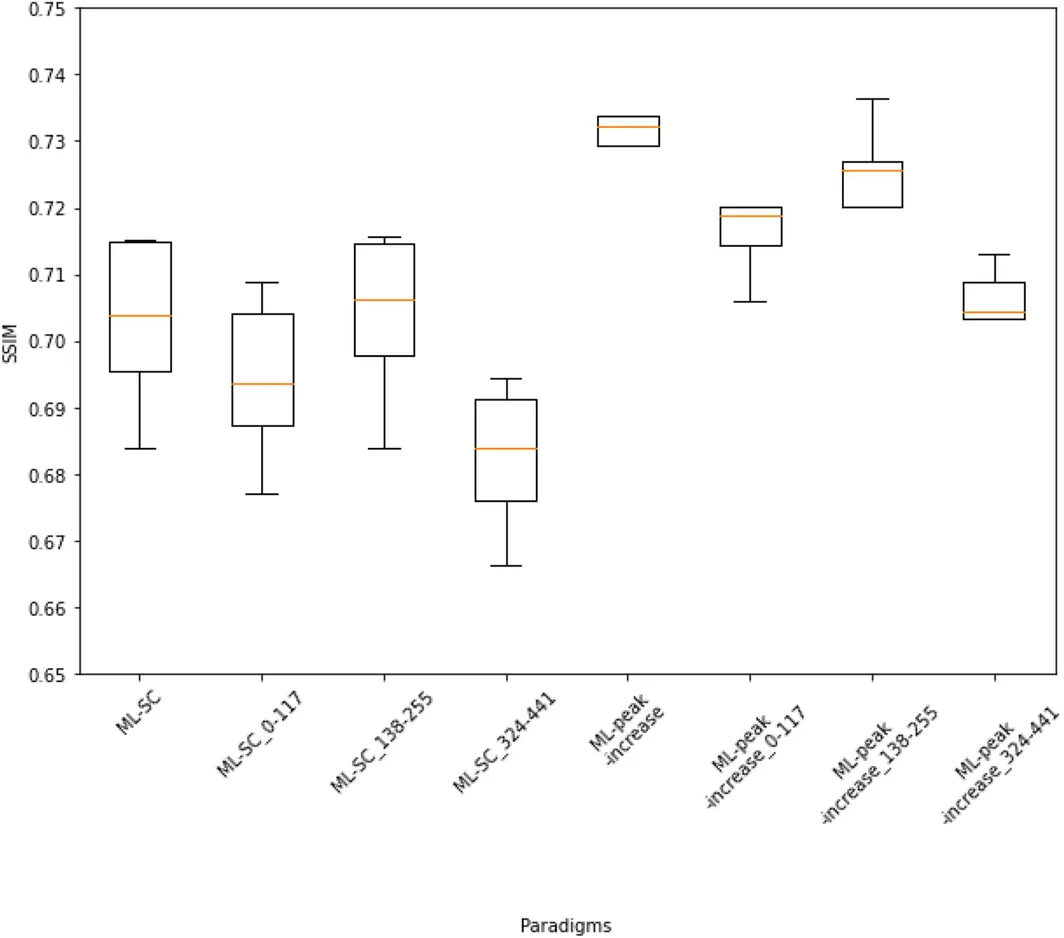
Box plot of mean SSIM of each paradigm versus seven task paradigms across the tested participants. The ML‐peak‐increase SSIM was significantly larger than the ML‐peak‐increase‐0–117, ML‐peak‐increase‐138–255 and ML‐peak‐increase‐324–441 SSIMs.

Each column in the box plot of Figure 7 represents the distribution of mean SSIM values for the activation maps generated from a RotF training scenario compared with the SC, SWG, R, AG and SCL task paradigms. The ON and PSL tasks were not considered because they were consistently inferior when compared with other RotF activation maps. In other words, the SSIM of the SC, SWG, R, AG and SCL tasks was consistently better or statistically equivalent when compared with other RotF training scenarios, as observed in Section 3.1.1. Moreover, SSIM was only calculated for participants who had a questionnaire score of 4 or above and head motion of less than 0.2 mm, as it was the threshold for which good quality RotF activation maps were produced (Refer to Section 3.1 for more). From Figure 7, the overall median of the ML‐peak‐increase training scenarios, comprising ML‐peak‐increase_0–117, ML‐peak‐increase_138–255 and ML‐peak‐increase_324–441 was higher than that of the ML‐SC training scenarios, comprising ML‐SC_0–117, ML‐SC_138–255, ML‐SC_324–441. The ML‐peak‐increase_138–255 training scenario, which utilised the segment found by SFFE to contribute most to RotF prediction, also had the highest overall median SSIM when compared to its counterparts, ML‐peak‐increase_0–117 and ML‐peak‐increase_324–441. While ML‐SC evaluated on shortened segments produced results that were inferior to ML‐peak‐increase, ML‐SC_138–255 also produced the highest overall median SSIM when compared to ML‐SC_0–117 and ML‐SC_324–441. The DICE results were consistent with the SSIM findings.

The participant‐level SSIM differed significantly between the paradigms (Friedman Test, $H\left(7\right)=684,p=0$). Post hoc testing revealed no significant difference between the participant‐level SSIM of the ML‐SC and ML‐SC‐138–255 paradigms. However, the participant‐level SSIM of the ML‐peak‐increase were found to be larger compared to the ML‐peak‐increase‐0–117, ML‐peak‐increase‐138–255 and ML‐peak‐increase‐324–441.

### 3.4 Rescan of Participants Using the Short Time Frame

A pilot study was conducted to investigate whether RotF generated activation maps corresponded to task activation with a shortened naturalistic stimulus. Three of the test participants were re‐scanned using only the short version of the quiz show (time frames 120 to 275; corresponding to 5 min and 10 s from 4 min to 9 min and 10 s of the original naturalistic stimulus). Additional time frames were added before the shortened naturalistic stimulus segment to allow for baseline fMRI signal stabilisation at the beginning of the fMRI scan, and extra time frames were added after the shortened segment to account for any delay in the hemodynamic response. The image acquisition parameters described in Section 2.3 were used. The acquired fMRI data from each participant were pre‐processed according to steps described in Section 2.4. The pre‐processed fMRI data were then constructed into test sets according to steps in Section 2.6.2 and evaluated.

#### 3.4.1 Correspondence Between Language Activation Maps

**FIGURE 8:**
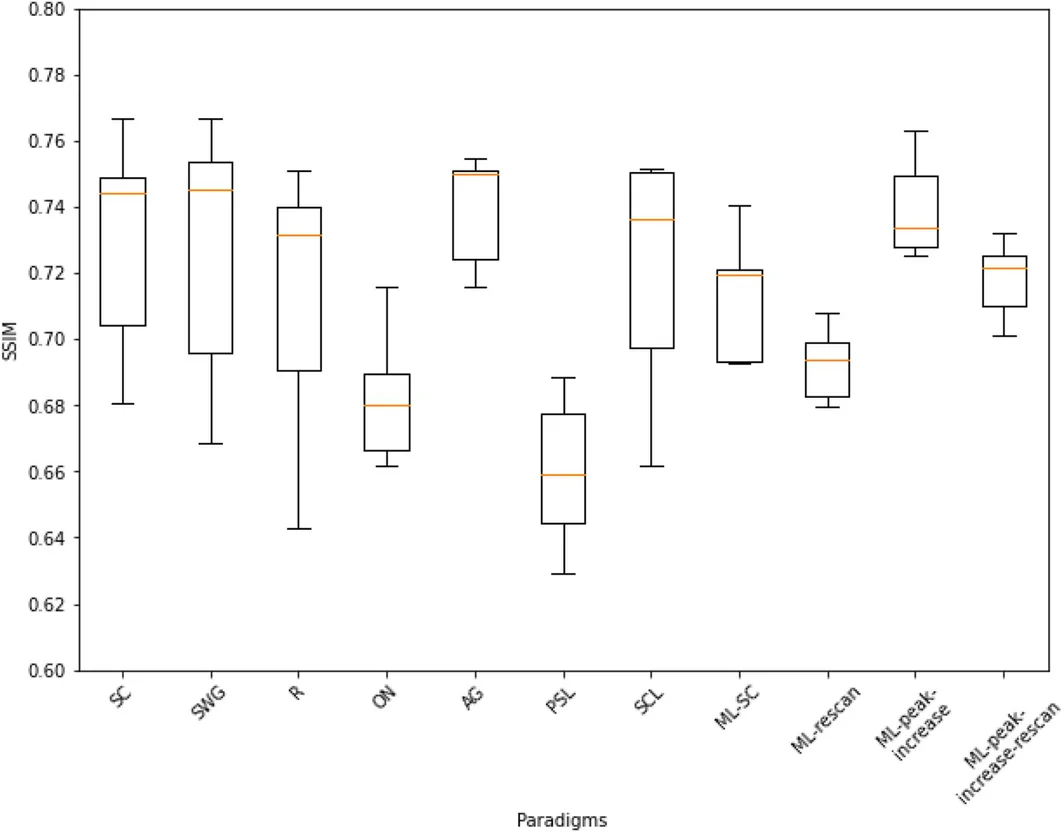
Box plot of mean SSIM of each paradigm versus seven task paradigms across all 3 participants that were re‐scanned using shortened naturalistic stimulus.

Figure 8 shows the box plots which represent the distribution of mean SSIM values when the activation maps of paradigms described in the *x*‐axis were compared to the seven language task activation maps. The mean SSIM for each comparison was derived by averaging across the three rescanned participants. The ML‐SC‐rescan column depicts results from the ML‐SC_138–255 model evaluated on the three re‐scanned participants. Similarly, the ML‐peak‐increase‐rescan column depicts results from the ML‐peak‐increase_138–255 model evaluated on the three rescanned participants.

Consistent with earlier results in Section 3.1.1, the overall median SSIM for the PSL task is the lowest between all language tasks and RotF training scenarios. This is observed through the lower position of the PSL box in Figure 8. While the overall median SSIM of both ML‐SC‐rescan and ML‐peak‐increase‐rescan is lower compared to their counterparts, namely the ML‐SC and ML‐peak‐increase training scenarios, they remained in the same range of the language tasks columns, suggesting a comparable result. The DICE results had larger variability but the trend was similar.

#### 3.4.2 Language Lateralisation

Laterality indices of the activation maps generated by the ML‐peak‐increase‐rescan training scenario were calculated for frontal and temporal regions and compared against those for each task paradigm (Table 3). LI dissimilarity was observed in the frontal ROI for the ON and PSL tasks. LI dissimilarity remained low in the temporal regions; however, some dissimilarity was observed for most of the tasks except for R and PSL. Interestingly, no LI dissimilarity was observed for the R task for both frontal and temporal regions.

**TABLE 3:** This table shows the activation threshold(s) for which the lowest percentage of participants present with laterality index dissimilarity between the activation maps produced by the ML‐peak‐increase‐rescan training scenario and seven task activation maps.

% (Threshold)	Frontal	Temporal
SC	0 (0.3)	33 (0.1, 0.15)
SWG	0 (0.25)	33 (0.1, 0.15, 0.2, 0.25)
R	0 (0.1, 0.15)	0 (0.1, 0.15)
ON	67 (0.1, 0.15, 0.2, 0.3)	33 (0.3)
AG	0 (0.3)	33 (0.1, 0.15, 0.3)
PSL	67 (0.1, 0.15, 0.2, 0.25, 0.3)	0 (0.25, 0.3)
SCL	0 (0.3)	33 (0.1, 0.2, 0.25, 0.3)

*Note:* Laterality index dissimilarity was computed for the frontal and temporal ROIs of the three rescanned participants.

#### 3.4.3 RotF Versus Task Activation Maps

**FIGURE 9:**
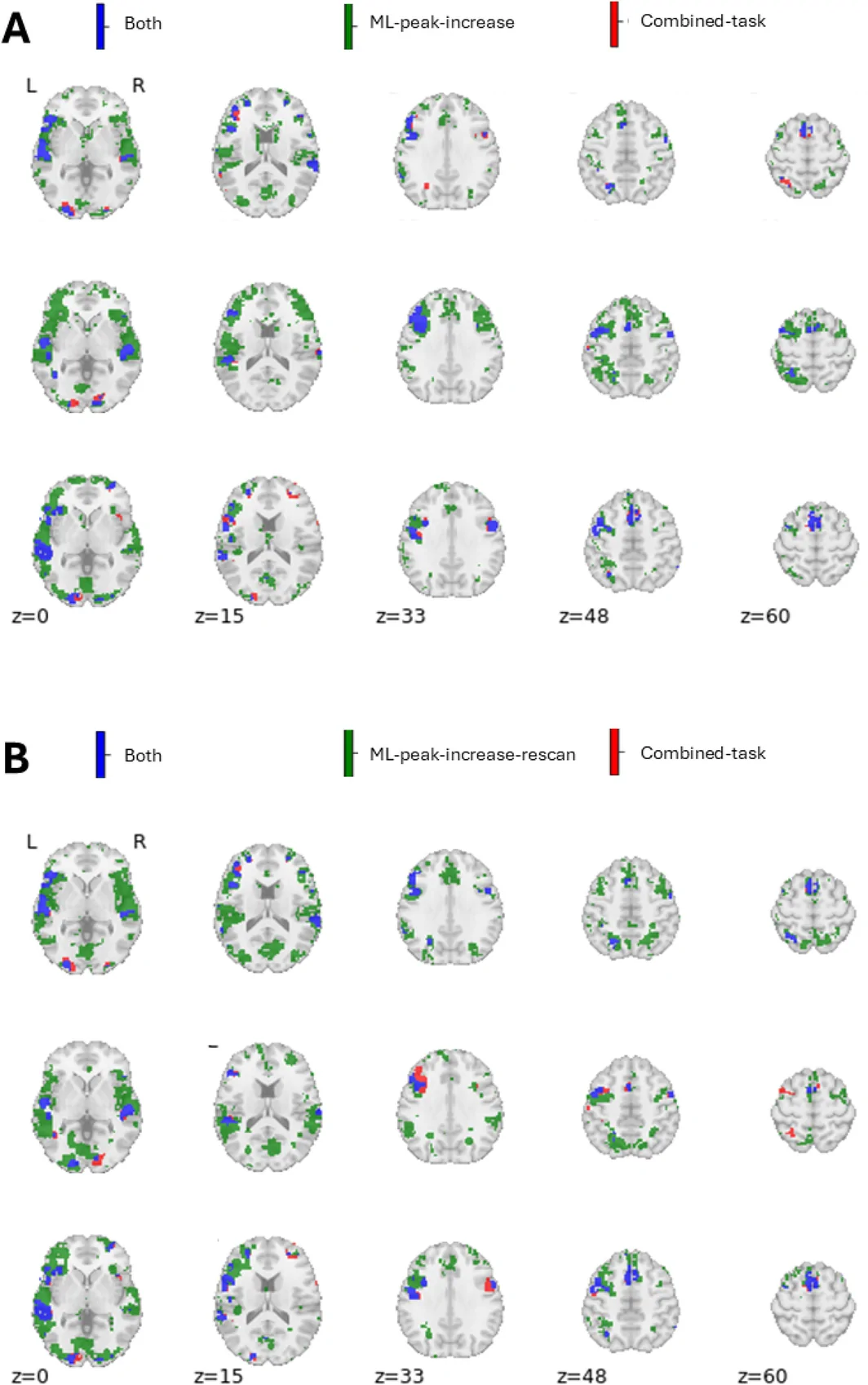
This figure shows the overlap between the combined task activation map versus the activation maps generated by ML‐peak‐(A) and ML‐peak‐increase‐rescan (B) (Blue—Overlap, Green—ML‐peak‐increase, Red—Combined‐task).

Figure 9 shows the overlaid activation maps of three re‐scanned participants scanned using the short time frame naturalistic stimulus. The activation maps produced with ML‐peak‐increase (A) and ML‐peak‐increase‐rescan (B) were superimposed on corresponding axial slices from the MNI brain template. This was overlaid with the combined task activation map generated by adding all task activation images. An activation threshold of 0.2 for both RotF generated and combined activation maps was applied for the purposes of illustration.

As in Figure 9, blue areas denote the overlap between the ML‐peak‐increase‐rescan and the combined task activation maps, green areas denote the activated areas only observed in the ML‐peak‐increase‐rescan, and the red areas denote those observed only in the combined task image. Language clusters including those in the left inferior frontal gyrus and bilateral posterior superior temporal gyrus were captured for all three participants. Bilateral temporal activation was identified as well.

## 4 Discussion

Our study addresses the challenges of analysing naturalistic fMRI data by decomposing 4D spatio‐temporal data into 1D time series classification using RotF, an algorithm previously found to perform well in the analysis of structured task‐based fMRI time series data (Kuan et al. 2024). RotF models were trained using 1D naturalistic fMRI time series from voxels activated in the language task‐based fMRI map, and the activation maps generated by RotF were concordant with task activation maps. In addition to temporo‐parietal activation, RotF activation maps showed activation clusters in the frontal regions, including Broca's area as observed in Figure 5. These activation patterns were observed to be consistent across all individuals. This suggests that RotF can be trained to map frontal, expressive language regions.

### 4.1 Role of Naturalistic fMRI

Naturalistic fMRI has been investigated for pre‐surgical language mapping patients with brain tumours but discussion of the design of the naturalistic stimulus has been limited. Tie et al. and Yao et al. (Tie et al. 2015; Yao et al. 2022) used a 7 min segment of a movie, ‘The Parent Trap’ which contained rich spoken language segments interleaved with segments of no speech. The authors found the paradigm to activate receptive language areas, and concluded the difficulty in passive watching paradigms to elicit activation in expressive language areas. Yao et al. (2022) suggested that phonological tasks such as the rhyming (R) task or antonym generation (AG) task may still be better suited for mapping frontal, expressive language regions. Although passive viewing paradigms often struggle to elicit robust activation in expressive language regions, our findings demonstrate that combining engaging television content with specially trained machine learning models can generate activation maps which highlight these regions effectively. This has important clinical implications as it provides a viable, less demanding alternative for patients who are unable to follow complex instructions associated with task fMRI scans. The findings of this study potentially expand access to accurate language mapping for individuals with cognitive or motor impairments. While ISC methods remain an established framework for analysing naturalistic fMRI data, methodological constraints such as reliance on predefined regions and a focus on group‐level analysis limit their effectiveness for participant‐specific language mapping, as highlighted in previous work (Kuan 2026).

### 4.2 Data Quality Control Measures for Naturalisatic fMRI

We found the incorporation of a post‐scan questionnaire and quantification of head motion provided an effective quality control step for acquiring naturalistic fMRI data, ensuring the acquired data were suitable for RotF‐based evaluation. Carefully designed post‐scan questionnaires can supplement in‐scanner monitoring of participant attention and are particularly valuable when additional measures such as eye‐tracking are not available. This helps determine whether participants have engaged in the naturalistic stimulus to a level that allows successful brain mapping, of particular importance for clinical utility. Patient engagement and motion can be highly variable and this approach enables clinicians or researchers to identify scans with sufficient quality for reliable language mapping, ultimately improving robustness and confidence in naturalistic fMRI–based assessment.

### 4.3 Where Is the Limit?

This study demonstrated that a 5 min 10 s segment identified by SFFE could produce language activation maps comparable to those from the full length, 15 min naturalistic stimulus. This finding greatly increases the feasibility of using naturalistic fMRI for language mapping in patients who cannot maintain attention for prolonged periods or struggle to tolerate long scans. The ability to achieve robust activation maps with a short, engaging naturalistic stimulus has the potential to expand access to pre‐surgical functional imaging, particularly for vulnerable or fatigued patient populations.

The RotF method does not take account of the temporal adjacency of the data, which may suggest that any 5 min segment could be chosen as a shortened protocol. While this may seem like a positive finding for naturalistic fMRI stimulus development, it does not take into consideration the temporal dependence of the hemodynamic response function. Future work should investigate this relationship to understand whether temporal dependence is implied via the hemodynamic response due to the stimulus. In addition, a 2 s TR was used in our protocol, which may be less sensitive to the rapid dynamics of naturalistic speech processing compared to a multiband‐accelerated sequence with TR of less than 800 ms (e.g., Human Connectome Project protocol). Future work should consider the use of smaller TRs to enable finer temporal sampling of language‐related neural activity.

### 4.4 Activation Maps Outside of Language Areas

While our study focused on a single naturalistic stimulus, our approach could be extended to other types of naturalistic stimuli to map other regions of the brain. It is important to note that good quality task activation maps or activation maps that target specific areas of interest in individuals are still an important precursor to good quality RotF models. Further work could evaluate unsupervised or model free methods in generating these activation maps. We used task activation maps as the ground truth in our study; while the RotF generated activation maps show strong correspondence with the SC, SWG, AG and SCL paradigms, they are weak when compared to the ON and PSL tasks. This could be because the RotF models were trained on the combined task activation map, which allows the RotF models to prioritise language regions that show consistent agreement across participants and tasks. An inevitable consequence is the reduced sensitivity to task‐specific sub‐networks close to pathological lesions potentially of interest for pre‐surgical planning. The sensitivity and the generalisability of RotF models remain a trade‐off, and future validation of RotF generated activation maps should include evaluation against language areas identified by direct cortical stimulation (Tie et al. 2015).

## 5 Conclusions

The research demonstrates effective mapping of both receptive and expressive language regions in the brain using naturalistic fMRI stimulus analysed using RotF. Our results show that RotF generated activation maps correspond with task fMRI activation patterns, particularly in the temporo‐parietal and frontal regions. Based on the original naturalistic fMRI stimulus, a shortened version was found to produce high‐quality activation maps, offering a patient‐friendly alternative to long fMRI scans for language mapping. Post‐scan questionnaires and a measure of head motion provide insight into naturalistic fMRI data quality. These findings suggest that naturalistic fMRI, when paired with an appropriate machine learning technique, holds significant promise for pre‐surgical language mapping and could improve clinical practices and workflows by offering an accessible and efficient method for visualising brain activation relating to language.

## Funding

This work was supported by the Australian Research Council (IC170100035).

## Conflicts of Interest

The authors declare no conflicts of interest.

## Data Availability

The data that support the findings of this study are available on request from the corresponding author. The data are not publicly available due to privacy or ethical restrictions.

## Appendix A Test set size for different participants (see Table A1)

**TABLE A1:** Number of 1D time series samples used from each participant. HC and EP mean healthy control epilepsy patient, respectively, and the associated number is the patient identifier from the dataset.

Participant ID	Number of 1D test samples
HC16	67,606
HC17	65,943
HC18	78,854
HC19	75,128
HC20	72,835
HC21	64,522
EP01	47,667
EP02	43,396
EP03	73,555
EP04	76,522
EP05	69,138
EP06	74,942
EP07	68,868
EP08	64,032
EP09	67,325
EP11	63,648
EP12	69,575
EP13	40,260
EP14	67,189
EP15	45,901
EP16	50,190
EP17	70,205
EP18	53,602
EP19	74,538
EP20	57,921
EP21	77,812
EP22	75,660
EP23	46,938
EP24	54,801
EP25	71,290
EP26	67,655
EP27	41,080
EP28	76,001
EP29	42,774
EP30	51,097
EP31	67,940

### Post‐Processing for fMRI Activation Maps to Account for SSIM Bias

The post‐processing steps applied to the task‐based and RotF generated activation maps were as follows. For each test participant, voxels containing values less than zero in each language task activation maps were converted zero. The average number of voxels converted to zero of all language tasks and test participants was calculated and saved. Then, all voxel values in each task activation map were min–max mapped to zero or one. The number of voxels with the smallest values in the RotF generated activation maps was converted to zero according to the average number of voxels converted to zero in the task activation maps. The RotF generated activation maps were then also min–max mapped to zero or one.

While SSIM is widely used to compare different images including medical images, the measure is susceptible to bias due to the image range, and bias correction is required. According to (Gourdeau et al. 2022), the SSIM formula is made up of several terms, including luminance, contrast, and structure. While contrast and structure components are insensitive to the image range, the luminance term remains sensitive to image range. Hence, we implemented the SSIM formula manually according to (Gourdeau et al. 2022) and varied the alpha value (which is a value associated with the luminance term within the SSIM formula) when comparing task activation maps versus RotF generated activation maps. An alpha value of 0.0001 was shown to give the best SSIM value.

## References

[hbm70638-bib-0001] Aliko, S. , J. Huang , F. Gheorghiu , S. Meliss , and J. I. Skipper . 2020. “A Naturalistic Neuroimaging Database for Understanding the Brain Using Ecological Stimuli.” Scientific Data 7, no. 1: 347. 33051448 10.1038/s41597-020-00680-2 PMC7555491

[hbm70638-bib-0002] Bartels, A. , and S. Zeki . 2004. “Functional Brain Mapping During Free Viewing of Natural Scenes.” Human Brain Mapping 21, no. 2: 75–85. 14755595 10.1002/hbm.10153 PMC6872023

[hbm70638-bib-0003] Benjamin, C. F. A. 2020. “Presurgical Language fMRI in Epilepsy: An Introduction.” In Translational Neuroscience of Speech and Language Disorders, 205–239. Springer International Publishing.

[hbm70638-bib-0004] Berl, M. M. , L. A. Zimmaro , O. I. Khan , et al. 2014. “Characterization of Atypical Language Activation Patterns in Focal Epilepsy.” Annals of Neurology 75, no. 1: 33–42. 24038442 10.1002/ana.24015 PMC4209919

[hbm70638-bib-0005] Binder, J. R. , S. J. Swanson , T. A. Hammeke , et al. 1996. “Determination of Language Dominance Using Functional MRI: A Comparison With the Wada Test.” Neurology 46, no. 4: 978–984. 8780076 10.1212/wnl.46.4.978

[hbm70638-bib-0006] Black, D. F. , B. Vachha , A. Mian , et al. 2017. “American Society of Functional Neuroradiology–Recommended fMRI Paradigm Algorithms for Presurgical Language Assessment.” American Journal of Neuroradiology 38, no. 10: E65–E73. 28860215 10.3174/ajnr.A5345 PMC7963630

[hbm70638-bib-0007] Bookheimer, S. 2007. “Pre‐Surgical Language Mapping With Functional Magnetic Resonance Imaging.” Neuropsychology Review 17: 145–155. 17484055 10.1007/s11065-007-9026-x

[hbm70638-bib-0008] Desmond, J. E. , J. M. Sum , A. D. Wagner , et al. 1995. “Functional MRI Measurement of Language Lateralization in Wada‐Tested Patients.” Brain 118, no. 6: 1411–1419. 8595473 10.1093/brain/118.6.1411

[hbm70638-bib-0009] Eickhoff, S. B. , M. Milham , and T. Vanderwal . 2020. “Towards Clinical Applications of Movie fMRI.” NeuroImage 217: 116860. 32376301 10.1016/j.neuroimage.2020.116860

[hbm70638-bib-0010] Fedorenko, E. , P.‐J. Hsieh , A. Nieto‐Castañón , S. Whitfield‐Gabrieli , and N. Kanwisher . 2010. “New Method for fMRI Investigations of Language: Defining ROIs Functionally in Individual Subjects.” Journal of Neurophysiology 104, no. 2: 1177–1194. 20410363 10.1152/jn.00032.2010 PMC2934923

[hbm70638-bib-0011] Ferri, F. J. 1994. “Comparative Study of Techniques for Large‐Scale Feature Selection.” In Machine Intelligence and Pattern Recognition, vol. 16, 403–413. Elsevier.

[hbm70638-bib-0012] Finn, E. S. , and P. A. Bandettini . 2021. “Movie‐Watching Outperforms Rest for Functional Connectivity‐Based Prediction of Behavior.” NeuroImage 235: 117963. 33813007 10.1016/j.neuroimage.2021.117963 PMC8204673

[hbm70638-bib-0013] Gorgolewski, K. J. , G. Varoquaux , G. Rivera , et al. 2015. “NeuroVault. Org: A Web‐Based Repository for Collecting and Sharing Unthresholded Statistical Maps of the Human Brain.” Frontiers in Neuroinformatics 9: 8. 25914639 10.3389/fninf.2015.00008 PMC4392315

[hbm70638-bib-0014] Gourdeau, D. , S. Duchesne , and L. Archambault . 2022. “On the Proper Use of Structural Similarity for the Robust Evaluation of Medical Image Synthesis Models.” Medical Physics 49, no. 4: 2462–2474. 35106778 10.1002/mp.15514

[hbm70638-bib-0015] Hasson, U. , R. Malach , and D. J. Heeger . 2010. “Reliability of Cortical Activity During Natural Stimulation.” Trends in Cognitive Sciences 14, no. 1: 40–48. 20004608 10.1016/j.tics.2009.10.011 PMC2818432

[hbm70638-bib-0016] Hinke, R. M. , X. Hu , A. E. Stillman , et al. 1993. “Functional Magnetic Resonance Imaging of Broca's Area During Internal Speech.” Neuroreport 4, no. 6: 675–678. 8347806 10.1097/00001756-199306000-00018

[hbm70638-bib-0017] Jääskeläinen, I. P. , M. Sams , E. Glerean , and J. Ahveninen . 2021. “Movies and Narratives as Naturalistic Stimuli in Neuroimaging.” NeuroImage 224: 117445. 33059053 10.1016/j.neuroimage.2020.117445 PMC7805386

[hbm70638-bib-0018] Kauppi, J.‐P. , J.‐P. Kauppi , J. Pajula , J. Niemi , R. Hari , and J. Tohka . 2017. “Functional Brain Segmentation Using Inter‐Subject Correlation in fMRI.” Human Brain Mapping 38, no. 5: 2643–2665. 28295803 10.1002/hbm.23549 PMC6867053

[hbm70638-bib-0019] Khosla, M. 2020. “A Shared Neural Encoding Model for the Prediction of Subject‐Specific fMRI Response.” In Medical Image Computing and Computer Assisted Intervention–MICCAI 2020: 23rd International Conference, Lima, Peru, October 4–8, 2020, Proceedings, Part VII 23, 539–548. Springer.

[hbm70638-bib-0020] Knecht, S. , A. Jansen , A. Frank , et al. 2003. “How Atypical Is Atypical Language Dominance?” NeuroImage 18, no. 4: 917–927. 12725767 10.1016/s1053-8119(03)00039-9

[hbm70638-bib-0021] Kuan, E. , V. Vegh , J. Phamnguyen , K. O'Brien , A. Hammond , and D. Reutens . 2024. “Language Task‐Based fMRI Analysis Using Machine Learning and Deep Learning.” Frontiers in Radiology 4: 1495181. 39664795 10.3389/fradi.2024.1495181 PMC11631583

[hbm70638-bib-0022] Kuan, E. Y. F. 2026. “Language Mapping Using Machine Learning and Deep Learning Methods With Naturalistic fMRI.” PhD thesis, The University of Queensland.

[hbm70638-bib-0023] Lahnakoski, J. M. , J. Salmi , I. P. Jääskeläinen , et al. 2012. “Stimulus‐Related Independent Component and Voxel‐Wise Analysis of Human Brain Activity During Free Viewing of a Feature Film.” PLoS One 7, no. 4: e35215. 22496909 10.1371/journal.pone.0035215 PMC3320648

[hbm70638-bib-0024] Löning, M. 2019. “Sktime: A Unified Interface for Machine Learning With Time Series.” arXiv Preprint arXiv:1909.07872.

[hbm70638-bib-0025] Norman, K. A. , S. M. Polyn , G. J. Detre , and J. V. Haxby . 2006. “Beyond Mind‐Reading: Multi‐Voxel Pattern Analysis of fMRI Data.” Trends in Cognitive Sciences 10, no. 9: 424–430. 16899397 10.1016/j.tics.2006.07.005

[hbm70638-bib-0026] Oota, S. R. 2019. “StepEncog: A Convolutional LSTM Autoencoder for Near‐Perfect fMRI Encoding.” In 2019 International Joint Conference on Neural Networks (IJCNN), 1–8. IEEE.

[hbm70638-bib-0027] Powell, H. W. R. , H. W. R. Powell , G. J. M. Parker , et al. 2007. “Abnormalities of Language Networks in Temporal Lobe Epilepsy.” NeuroImage 36, no. 1: 209–221. 17400477 10.1016/j.neuroimage.2007.02.028

[hbm70638-bib-0028] Power, J. D. , K. A. Barnes , A. Z. Snyder , B. L. Schlaggar , and S. E. Petersen . 2012. “Spurious but Systematic Correlations in Functional Connectivity MRI Networks Arise From Subject Motion.” NeuroImage 59, no. 3: 2142–2154. 22019881 10.1016/j.neuroimage.2011.10.018 PMC3254728

[hbm70638-bib-0029] Seghier, M. L. , F. Lazeyras , A. J. Pegna , et al. 2004. “Variability of fMRI Activation During a Phonological and Semantic Language Task in Healthy Subjects.” Human Brain Mapping 23, no. 3: 140–155. 15449358 10.1002/hbm.20053 PMC6871802

[hbm70638-bib-0030] Simony, E. , and C. Chang . 2020. “Analysis of Stimulus‐Induced Brain Dynamics During Naturalistic Paradigms.” NeuroImage 216: 116461. 31843711 10.1016/j.neuroimage.2019.116461 PMC7418522

[hbm70638-bib-0031] SKTIME . 2023. “Time Series Classification.” https://sktime‐backup.readthedocs.io/en/v0.13.4/api_reference/classification.html.

[hbm70638-bib-0032] Sonkusare, S. , M. Breakspear , and C. Guo . 2019. “Naturalistic Stimuli in Neuroscience: Critically Acclaimed.” Trends in Cognitive Sciences 23, no. 8: 699–714. 31257145 10.1016/j.tics.2019.05.004

[hbm70638-bib-0033] Spiers, H. J. , and E. A. Maguire . 2007. “Decoding Human Brain Activity During Real‐World Experiences.” Trends in Cognitive Sciences 11, no. 8: 356–365. 17618161 10.1016/j.tics.2007.06.002

[hbm70638-bib-0034] Tie, Y. , L. Rigolo , A. Ozdemir Ovalioglu , et al. 2015. “A New Paradigm for Individual Subject Language Mapping: Movie‐Watching fMRI.” Journal of Neuroimaging 25, no. 5: 710–720. 25962953 10.1111/jon.12251 PMC4537682

[hbm70638-bib-0035] Vanderwal, T. , J. Eilbott , and F. X. Castellanos . 2019. “Movies in the Magnet: Naturalistic Paradigms in Developmental Functional Neuroimaging.” Developmental Cognitive Neuroscience 36: 100600. 30551970 10.1016/j.dcn.2018.10.004 PMC6969259

[hbm70638-bib-0036] Vodrahalli, K. , P.‐H. Chen , Y. Liang , et al. 2018. “Mapping Between fMRI Responses to Movies and Their Natural Language Annotations.” NeuroImage 180: 223–231. 28648889 10.1016/j.neuroimage.2017.06.042 PMC5742073

[hbm70638-bib-0037] Yao, S. , L. Rigolo , F. Yang , et al. 2022. “Movie‐Watching fMRI for Presurgical Language Mapping in Patients With Brain Tumour.” Journal of Neurology, Neurosurgery & Psychiatry 93, no. 2: 220–221. 34172562 10.1136/jnnp-2020-325738 PMC8709882

